# Optical Signatures of Dirac Electrodynamics for hBN-Passivated Silicene on Au(111)

**DOI:** 10.1021/acs.nanolett.1c01440

**Published:** 2021-06-07

**Authors:** Jakob Genser, Daniele Nazzari, Viktoria Ritter, Ole Bethge, Kenji Watanabe, Takashi Taniguchi, Emmerich Bertagnolli, Friedhelm Bechstedt, Alois Lugstein

**Affiliations:** † Institute of Solid State Electronics, Technische Universität Wien, Gußhausstraße 25-25a, 1040 Vienna, Austria; ‡ Infineon Technologies Austria AG, Siemensstraße 2, 9500 Villach, Austria; § Research Center for Functional Materials, National Institute for Materials Science, 1-1 Namiki, Tsukuba 305-0044, Japan; ⊥ International Center for Materials Nanoarchitectonics, National Institute for Materials Science, 1-1 Namiki, Tsukuba 305-0044, Japan; ∥ IFTO, Friedrich Schiller Universität, Max-Wien Platz 1, 07743 Jena, Germany

**Keywords:** silicene, Au(111), passivation, optical properties, Dirac electrodynamics, Raman, differential reflectance spectroscopy

## Abstract

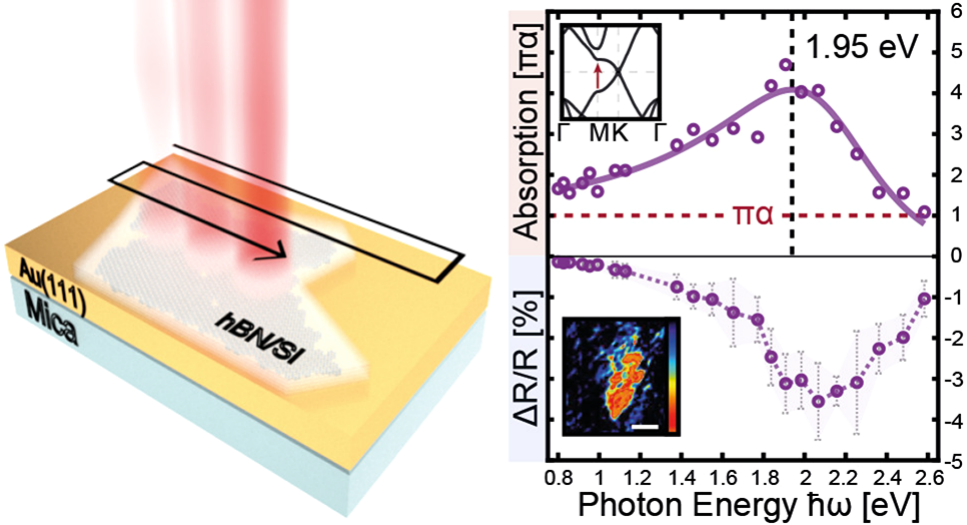

The allotropic affinity for bulk silicon and unique electronic and optical properties make silicene a promising candidate for future high-performance devices compatible with mature complementary metal–oxide–semiconductor technology. However, silicene’s outstanding properties are not preserved on its most prominent growth templates, due to strong substrate interactions and hybridization effects. In this letter, we report the optical properties of silicene epitaxially grown on Au(111). A novel in situ passivation methodology with few-layer hexagonal boron nitride enables detailed ex situ characterization at ambient conditions via μ-Raman spectroscopy and reflectance measurements. The optical properties of silicene on Au(111) appeared to be in accordance with the characteristics predicted theoretically for freestanding silicene, allowing the conclusion that its prominent electronic properties are preserved. The absorption features are, however, modified by many-body effects induced by the Au substrate due to an increased screening of electron–hole interactions.

## Introduction

Since its discovery, graphene has been the subject of comprehensive research primarily due to its outstanding electronic^1,2^ and optical properties.^3,4^ However, because of poor integration of graphene into mature complementary metal–oxide–semiconductor technology, the realization of widespread applications is limited thus far. Therefore, large interest for silicon (Si)-compatible 2D materials has been generated.^5^ In particular, the group IV 2D allotropes silicene, germanene, and stanene hold great promise for a variety of novel applications such as high-performance nanoelectronics or topological and quantum devices.^6^ Furthermore, their allotropic affinity with bulk Si possibly enables a more straightforward integration into existing semiconductor technology. Notably, silicene combines ultrahigh carrier mobility^7,8^ with a tunable band character^9^ and band gap, which appeared to be sensitive to substrate interaction, surface chemistry, and spin–orbit coupling.^9,10^ It was predicted that the electronic and optical properties of freestanding silicene closely resemble those of graphene, exhibiting a Dirac cone and Dirac electrodynamics,^11−14^ independent of the degree of sp^2^ and sp^3^ hybridization and the amount of sheet buckling.^11^

Due to the intrinsic instability of silicene, its synthesis requires ultrahigh vacuum (UHV) conditions and a supporting substrate.^8,15^ However, for Ag, the most prominent growth substrate of silicene,^16^ the electronic and optical properties of freestanding silicene are not preserved,^14,17,18^ due to either strong interactions with the substrate^17,19^ or a change in the atomic geometry.^20−22^ Among others, Au(111) was proposed to be a promising candidate for the stabilization of silicene.^23^ Scanning tunneling microscopy investigations revealed the hexagonal silicene structure on top of Au(111), however, with an increased lattice constant compared to that of freestanding silicene.^24^ Further, low interaction of the Si atoms with the Au substrate was demonstrated.^25^ Additionally, recent angle-resolved photoemission spectroscopy investigations proved the preservation of the Dirac cone for silicene grown on Au(111).^26^

In this letter, we show for the first time experimentally determined optical properties of silicene grown on Au(111). Passivation with hexagonal boron nitride (hBN) enabled detailed analysis of the encapsulated silicene layer at ambient conditions. Raman and reflectance measurements revealed an intact strained silicene layer on top of the Au(111) substrate, with the exceptional optical and electronic properties of freestanding silicene preserved.

## Results and Discussion

For the synthesis of silicene, a monolayer of Si is deposited on a Au(111) substrate at 533 K under UHV conditions, as described in detail in the Methods section. Due to the intrinsic instability of silicene, subsequent ex situ characterization requires an effective passivation without destroying its structural integrity. Inert 2D materials such as graphene or hBN have already been proven to be excellent candidates for the passivation of environmentally unstable materials.^27−29^ For graphene, we demonstrated recently that it is capable of forming an effective barrier against oxidizing species, while preserving the structural integrity of a subjacent silicene layer on Ag(111).^29^ In this study, silicene was passivated in situ directly after the growth using few-layer hBN, which has already been proven to be a suitable passivation for silicene^30^ and germanene.^31^ The intrinsic transparency of hBN with a wide band gap of 5.95 eV^32^ enables direct access to the optical properties of the subjacent silicene layer over a wide spectral range. Furthermore, the absence of Raman-active modes of hBN in the fingerprint region of silicene facilitates the evaluation of ex situ Raman measurements.

**Figure 1:**
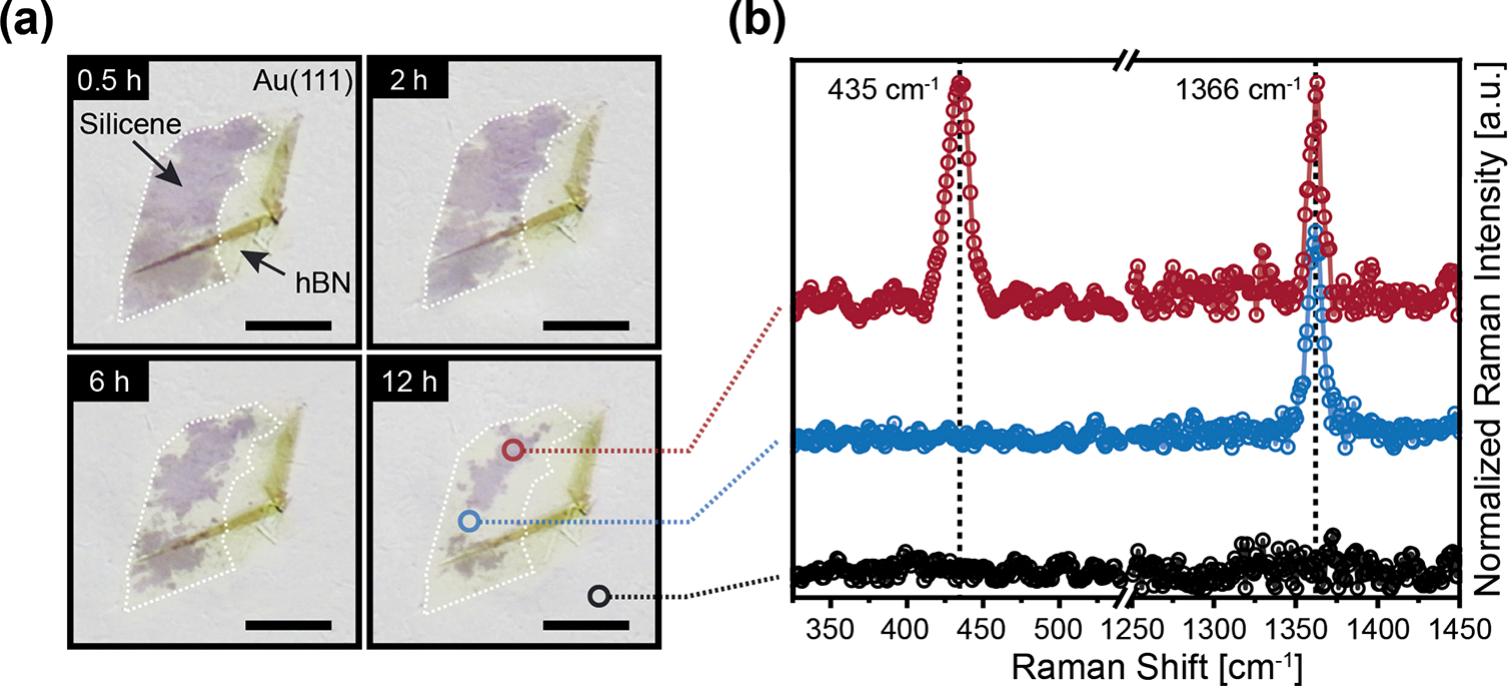
Optical micrographs of passivated silicene and correlated Raman characteristics. (a) Optical microscopy images of an hBN-passivated silicene sheet on Au(111), illustrating the degradation process of silicene over several hours. The dashed white line indicates the extent of the silicene layer after removal from the UHV growth chamber (scale bar 10 μm). (b) Raman spectra obtained at the positions marked in (a) of passivated silicene (red), degraded silicene under hBN (blue), and degraded silicene on top of Au(111) (black).

Figure 1a illustrates optical microscope images of passivated silicene on Au(111), revealing a clearly observable layer beneath the hBN flake. It is well-known that certain 2D materials with thicknesses in the sub-nanometer regime can exhibit strong optical responses at specific energy ranges, which is also expected for silicene.^11−14^ This phenomenon enables the degradation of the silicene layer to be monitored by optical means. Starting from the hBN edges, silicene slowly degrades as oxidizing species diffuse under the passivation layer. The stability of the passivated silicene layer at ambient conditions appeared to depend critically on the quality of the hBN capping flakes and the in situ exfoliation processing. The initial lack of optical contrast under certain areas of the hBN passivation does not indicate a bare Au(111) substrate but is a consequence of an already degraded silicene layer due to imperfections of the hBN capping. Similar observations have been made when passivating silicene on Ag(111) with few-layer graphene using the same technique.^29^ Notably, traces of intact silicene in the center of an hBN flake can even be detected after several days in ambient atmosphere.

Figure 1b shows the Raman signal measured at three points marked in the lower right plot of Figure 1a, for an intact (red) and a degraded (blue) silicene layer below hBN as well as the bare Au(111) substrate (black). In addition to the characteristic Raman peak of hBN at ≈1366 cm^–1^,^33^ an additional Raman feature at ≈435 cm^–1^ is observed only at the grayish areas. This peak assigned to silicene appears red-shifted compared to that of the freestanding silicene^34^ due to the larger lattice constant (4.1 Å) of silicene on Au(111),^24^ in good agreement with theoretical predictions.^24,35^ It can be excluded that the vibrational properties of silicene are influenced by the hBN capping, as they remain unchanged when using graphene as passivation layer (see Supporting Information Figure S1).

**Figure 2:**
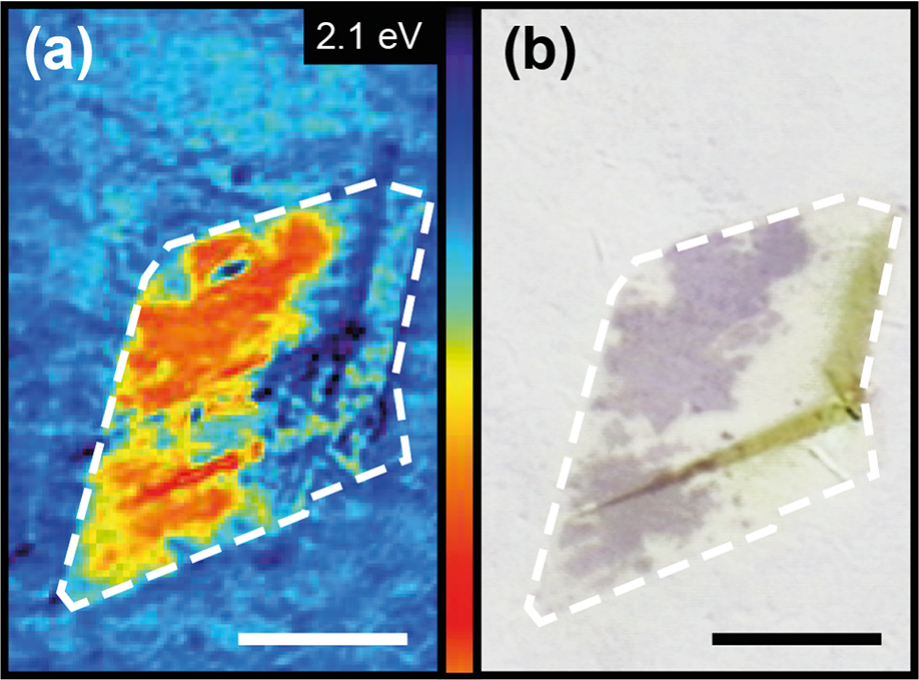
High-resolution μ-reflection mapping and optical image of an hBN-passivated silicene layer on Au(111). (a) Reflection mapping of an hBN-passivated silicene layer on Au(111), recorded at a laser energy of 2.1 eV (scale bar 10 μm, logarithmic *z* scale in detected counts [au]). (b) Optical microscopy image taken immediately after the reflectance measurement (scale bar 10 μm). The dashed white border indicates the edge of the hBN capping flake.

To gain access to the optical properties, normal incident reflectance measurements were performed,^36^ which have already been proven to be suitable for graphene^37^ and further 2D materials.^38^ However, the spatial limitation of the silicene layer and the presence of additional phases^24,26^ do not allow for accurate large-area measurement techniques. The reflection mapping illustrated in Figure 2a was achieved by highly resolved area scans with a small spot size at a laser energy of 2.1 eV. As expected, the Au(111) substrate shows a quite homogeneous reflection pattern. In contrast, for certain areas below the hBN passivation layer, a clear decrease in reflectance corresponding to a higher absorption can be observed. When the obtained spatial distribution of the reflectance was compared with the optical microscopy image in Figure 2b, the reduced reflectance can be clearly assigned to the silicene layer. The confocal setup and tunable laser source allow one to record spatial distributions of the reflectance at specific laser energies in the energy regime of 0.8–2.58 eV.

The relative change of the reflectance caused by the silicene layer is defined by
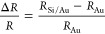
1where *R*_Si/Au_ and *R*_Au_ denote the average reflectance of Au with and without a superjacent silicene layer.

Even though hBN is optically inactive in the energy range under investigation, a change in contrast induced by interference of thin film multilayers may be expected for layers at certain thicknesses.^33^ However, for the thin hBN passivation layers used in this study, no significant change in reflectance has been detected. This finding is in agreement with previous reports on 2D heterostructures, where thin hBN sheets only lead to a minor increase of the relative change of the reflectance due to interlayer coupling effects.^39,40^ Consequently, there should be no influence on the shape and position of the observed silicene-related reflectance features, although the determined values could be slightly overestimated.

**Figure 3:**
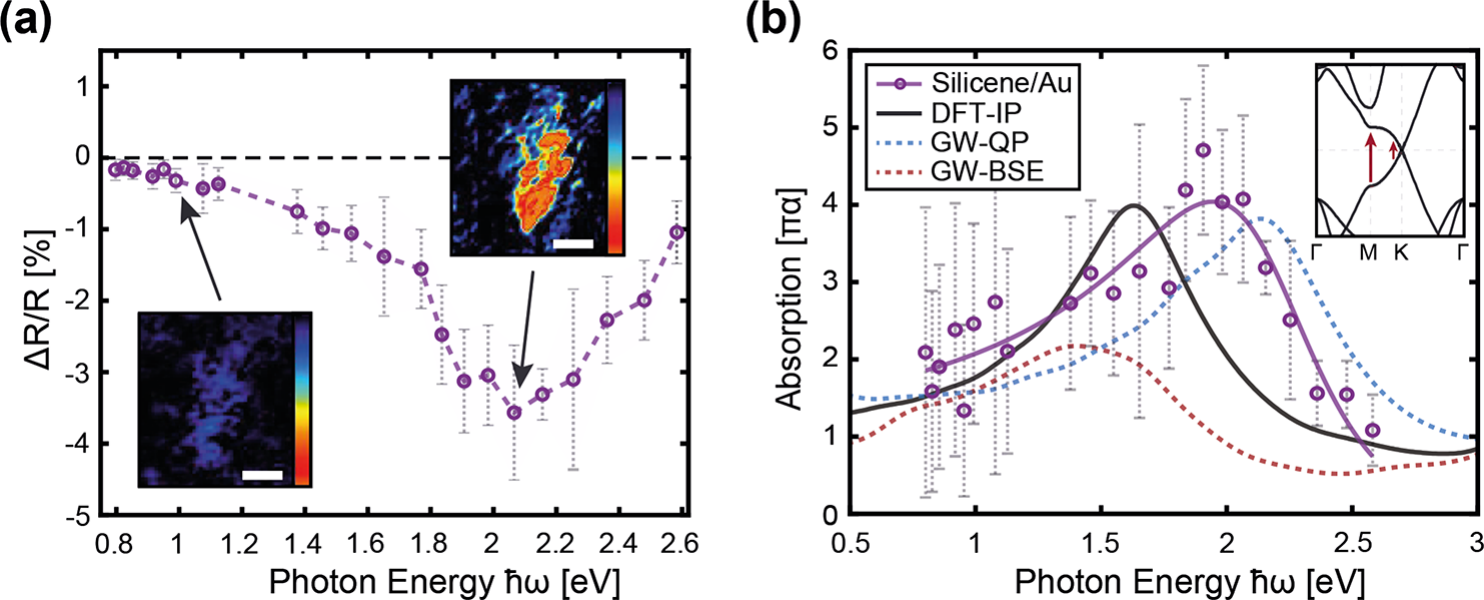
Reflection and absorption spectra of silicene on Au(111). (a) Fractional change in reflectance of silicene on Au(111), determined according to formula 1 from experimental reflection mappings with a clear minimum at ≈2.1 eV. The insets show Δ*R*/*R* mappings at the marked laser energies (scale bar 5 μm, logarithmic *z* scale from −3.5 to 0%). (b) Absorption spectrum of silicene on Au(111), calculated from the Δ*R*/*R* values with formula 2 (solid purple line) and previously calculated absorption spectra of freestanding silicene, adapted with permission from ref (14) (copyright 2018 American Physical Society): IP approximation using the DFT eigenvalues (solid black line), GW approximation including QP corrections (dashed blue line), and additionally solving the BSE considering excitonic effects (dashed red line). The inset illustrates a schematic band diagram of silicene^41,42^ with the relevant possible optical transitions indicated.

The resulting wavelength-dependent change in reflectance caused by the silicene layer is illustrated in Figure 3a. The relative reflectance change due to the presence of the silicene layer in the infrared region is only minor and progressively decreases for lower energies. This is the expected behavior for silicene on metallic substrates,^13,18^ and it is in accordance with its absorbance in this energy regime, being characterized by a universal value of πα = 0.0229.^11−13^ Although the experimental determination of such a small effect is challenging, it was still possible to clearly identify the silicene sheets in the reflection mapping, as illustrated in the lower inset of Figure 3a. At higher photon energies, the contrast becomes more pronounced and a reflection minimum can be clearly observed at ∼2.1 eV. Additional large-area reflectance measurements of the Au(111) substrate, with and without an unprotected silicene layer, clearly revealed that this feature can only be observed for an intact silicene layer (see Supporting Information Figure S2).

The reflection minimum can apparently be associated with the absorption peak proposed for freestanding silicene, corresponding to the van Hove singularities of the joint density of states.^11,12^ Assuming normal incidence in a three-layer system, it is possible to determine the absorbance and the real part of the sheet conductivity of a 2D material directly from reflectance measurements.^13,37^ Since the contribution of the imaginary part of the sheet conductivity of silicene and the hBN encapsulation proved to be negligible, one can calculate the absorbance of the isolated silicene layer as
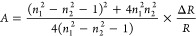
2where *n*_1_ is the refractive index and *n*_2_ is the extinction coefficient of the underlying Au substrate. Since changes in the optical constants of the Au substrate directly influence the calculated absorption of the isolated silicene sheet, the presence of the degraded silicene layer also needs to be considered. The additional unprotected silicene layer, however, proved to induce no distinct differences compared to the pristine Au substrate (see Supporting Information Figure S3).

The resulting absorbance of the quasi-freestanding silicene sheet is illustrated in Figure 3b, with the *n*_1_ and *n*_2_ values of Au taken from previous studies of Johnson et al.^43^ By extracting the pure optical influence of the Au substrate via formula 2, a shift of the prominent absorption peak to lower photon energies and an increase of the values in the infrared region in the absorption spectrum can be observed. The measured absorption curve is compared with absorption spectra previously calculated for freestanding silicene within different approximations on the many-body effects, quasiparticle (QP) renormalization and electron–hole (e–h) attraction on top of the independent particle (IP) approximation using the electronic structure of the density functional theory (DFT) model. QP effects are treated applying Hedin’s GW approximation on the exchange-correlation self-energy, whereas the excitonic effects are included by solving the Bethe–Salpeter equation (BSE) for the optical response function.^44^ Overall, the absorption closely resembles the predicted absorption of freestanding silicene.^11−14^ In particular, the formation of a low-energy absorption edge is an unambiguous difference from the absorbance expected from bulk silicon^45,46^ and also from that of other reported silicon allotropes.^47,48^ This difference can only be explained by the allowed π–π* transitions between the linear bands present in sp^2^-bonded silicene. The absorption in the infrared region near 1 eV is expected to be larger than πα, as an absorption of πα is only anticipated at the limit of ω → 0. At slightly higher photon energies, the absorbance is expected to increase as ≈ω^2^, due to the deviation of the higher interband energies from the linearity of the Dirac cone.^11^

Furthermore, the distinct absorption peak with a maximum at about 1.95 eV appears located between the theoretical peaks in the IP and GW-QP description of stand-alone silicene but is blue-shifted by roughly 400 meV compared to the predicted peak including QP and excitonic effects. The pronounced asymmetry of the absorption feature can be related to a Fano line shape,^49^ in agreement with the interpretation in the case of graphene.^50,51^ As already mentioned above, this absorption feature can be correlated to the van Hove singularity of the joint density of states.^11,12^ More precisely, it has to be identified with the *M*_1_ saddle-point van Hove singularity at the *M* point of freestanding silicene, caused by the π–π* interband transitions schematically illustrated in the inset of Figure 3b.^11,12^ While the band linearity along the KΓ line in the Brillouin zone extends toward 5 eV, along the KMΓ near M, the interband structure exhibits a saddle point giving rise to the observed absorption peak.^11−13^ Additionally, the absorption peak is modified by quasiparticle and excitonic effects with a tendency for compensation. This interpretation is in qualitative agreement with findings for graphene with a saddle point close to 4.2 eV or even higher energies, dependent on the substrate or treatment of the many-body effect.^37,50,52,53^ Furthermore, van Hove singularities with a minimum character and dipole-allowed optical transitions appear at much higher photon energies toward 4 eV in the interband structure near M and Γ,^12,16^ which are, however, outside the studied energy range. Hence, we conclude that electronic and optical properties of the Au-supported silicene remain predominantly intact but exhibit a blue shift of the absorption peak caused by the π–π* interband transitions near the M point.

The influence of the hBN passivation and implemented measurement technique, as discussed above, provides no satisfactory explanation for the observed shift of the absorption peak toward higher energies. Since for silicene on Au(111) an increased lattice parameter^24^ is reported, the effects on the absorption induced by biaxial tensile strain have to be considered. It was predicted that the Dirac cone in silicene is preserved even for high biaxial tensile strain.^35^ At the anticipated strain level, a noticeable red shift is expected for the absorption peak located toward 4 eV; however, the investigated peak caused by the π–π* interband transitions is predicted to experience no significant displacement.^54,55^

A similar blue shift was also reported for graphene on metallic substrates with respect to the position of the absorption feature on insulating substrates due to saddle-point excitons.^56−58^ This effect has been traced back to the increased screening of the e–h attraction by the metallic support, so that the compensation of the QP blue shift by the exciton-mediated red shift is suppressed, resulting in a net blue shift.^50,56^ The three theoretical absorption curves of freestanding silicene in Figure 3b show the same compensation tendencies, with respect to the peak position of the absorption feature. However, since the optical and electronic properties of silicene closely resemble those of graphene, only scaled by the Fermi velocities of the linear bonds,^12^ it is reasonable to assume that similar screening effects also occur for silicene on metallic substrates. Most probably, the supporting Au(111) substrate induces strong image potential effects, resulting in an increased screening in the silicene overlayer. Such increased screening influences both QP and excitonic effects. However, the effect on the QP interband energy remains small, whereas it is more pronounced for the screened e–h attraction, which is directly reduced by the additional screening. The accompanying suppression of the excitonic effects results in a reduced excitonic red shift and would explain the observed net shift of the absorption peak toward higher energies. Therefore, we conclude that the observed blue shift of the prominent absorption peak at ≈1.95 eV is apparently due to the increased screening of e–h interaction in silicene on the Au substrate.

## Conclusion

Summarizing, we investigated the optical properties of silicene grown on a Au(111) substrate. An hBN passivation layer was used to stabilize the silicene structure, enabling analysis of its properties at ambient conditions. Optical and Raman investigations identified the studied layer as the recently on Au(111) reported hexagonal silicene structure.^24^ The absorption spectrum, derived from μ-reflectance measurements, closely resembles the predicted absorption of freestanding silicene. At a photon energy of about 1.95 eV, an absorption feature corresponding to the van Hove singularity of the joint density of states can be observed. Its peak position is apparently modified by many-body effects, induced by the Au substrate. The presented work gives clear experimental evidence that the optical and electronic properties of silicene are preserved on a Au(111) substrate.

## Methods

The samples were prepared under UHV conditions at a base pressure of 5 × 10^–11^ mbar. A 300 nm thick Au(111) layer on mica (MaTecK) was used as a substrate, which was cleaned through several cycles of Ar^+^ sputtering (1 keV, 5 min), followed by subsequent annealing at 770 K for 20 min. Si was deposited at 533 K for 50 min with an evaporation rate of ≈0.02 ML/min from a Si rod via electron beam evaporation (EBE-1, SPECS). The substrate temperature during the growth and annealing was monitored by an infrared pyrometer with a accuracy of ±2 K. Immediately after the growth, the samples were passivated in situ by exfoliated few-layer hBN in a dedicated UHV chamber, directly connected to the evaporation chamber.^29^ For full details of the implemented passivation technique, see the Supporting Information.

Ex situ Raman analysis in back-scattering geometry was implemented using a confocal μ-Raman setup (Alpha300, WITec). A frequency-doubled Nd:YAG laser emitting linearly polarized light at λ = 532 nm served as an excitation source, and the beam was focused on the sample via a Nikon 100× objective (NA = 0.9, WD = 0.23 mm), allowing a minimal spot size of ≈750 nm. To gain access to the optical properties of silicene via reflectance measurements at different wavelengths, white light from a broadband laser source (SuperK Extreme, NKT) was used. The light was coupled to a monochromator (SuperK Select, NKT) including three acoustic-optical tunable filters, enabling output of monochromatic light in the visible (λ = 450–700 nm), near-infrared (λ = 600–900 nm), and infrared (λ = 1100–2000 nm) spectra. The output of the monochromator was coupled into a WITec Alpha300 and focused on the surface through a Nikon 100× objective (NA = 0.9, WD = 0.23 mm), while passing a 50:50 beam splitter. The reflected light was detected in the visible (Andor iDus 401) or infrared light spectrum (Andor IDus InGaAs). All measurements were performed at ambient conditions, and the output power was chosen to have negligible laser heating effects on the silicene layer.

## Supporting Information Available

Comparison of the Raman signature of silicene grown on Au(111) dependent on the passivation material (Figure S1), reflectance spectrum (Figure S2), real and imaginary parts of the refraction index (Figure S3) for the Au(111) substrate with and without an oxidized silicene layer, and full detail of the implemented passivation technique (Figure S4) (PDF)
