# Supplementary material for: Optical Signatures of Dirac Electrodynamics for hBN-Passivated Silicene on Au(111)
Source: Nano Lett. 2021 Jun 7;21(12):5301–7. doi: 10.1021/acs.nanolett.1c01440 (PMC8227485; doi:10.1021/acs.nanolett.1c01440)
Supplement: Supplementary file 1 — nl1c01440_si_001.pdf [file nl1c01440_si_001.pdf]

Supporting Information

Optical Signatures of Dirac Electrodynamics for hBN Passivated Silicene on Au(111)

Jakob Genser,[†,⊥] Daniele Nazzari,[†,⊥] Viktoria Ritter,[†,⊥] Ole Bethge,[‡,†] Kenji Watanabe,[¶] Takashi Taniguchi,[§] Emmerich Bertagnolli,[†] Friedhelm Bechstedt,[∥] and Alois Lugstein[*,†]

†*Institute of Solid State Electronics, Technische Universität Wien, Gußhausstraße 25-25a, 1040 Vienna Austria*

‡*Infineon Technologies Austria AG, Siemensstraße 2, 9500 Villach, Austria*

¶*Research Center for Functional Materials, National Institute for Materials Science, 1-1 Namiki, Tsukuba 305-0044, Japan*

§*International Center for Materials Nanoarchitectonics, National Institute for Materials Science, 1-1 Namiki, Tsukuba 305-0044, Japan*

∥*IFTO, Friedrich Schiller Universität, Max-Wien Platz 1, 07743 Jena, Germany*

⊥*Contributed equally to this work*

E-mail: alois.lugstein@tuwien.ac.at

1. Vibrational properties of silicene passivated with graphene and hBN respectively

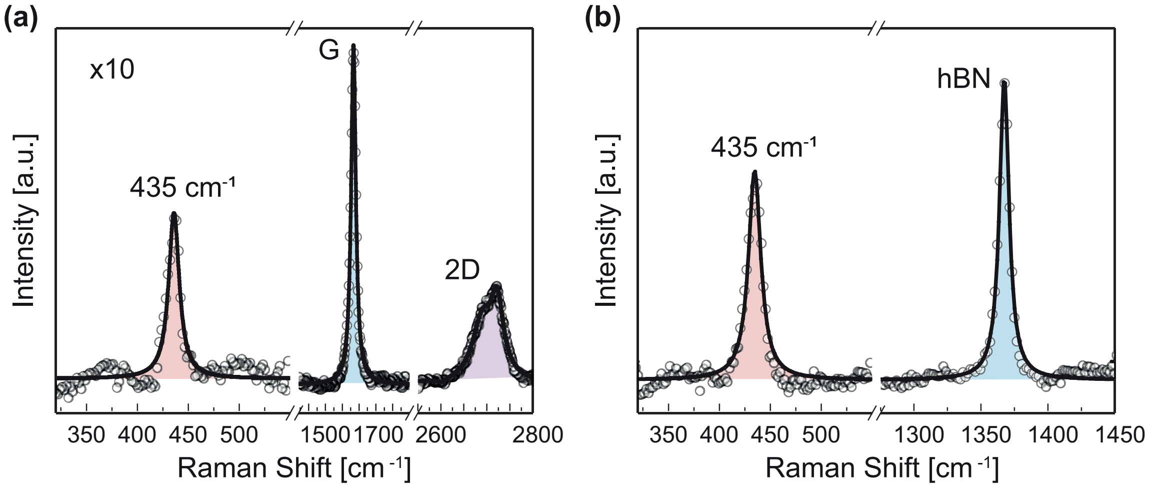

Figure S1: Vibrational properties of the silicene layer for different passivation materials. Raman spectrum of silicene grown on Au(111) passivated by (a) few-layer graphene and (b) few-layer hBN.

2. Optical properties of the Au(111) substrate

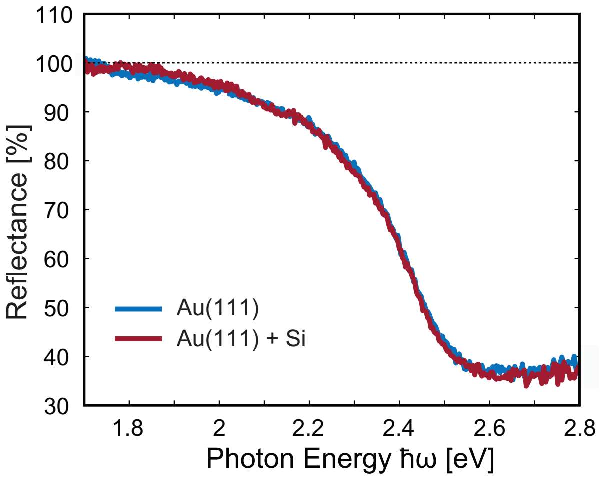

Figure S2: Ex-situ large area reflectance spectra of (a) the pristine Au(111) substrate and (b) the same Au(111) substrate with 1 ML silicene which degraded under ambient conditions.

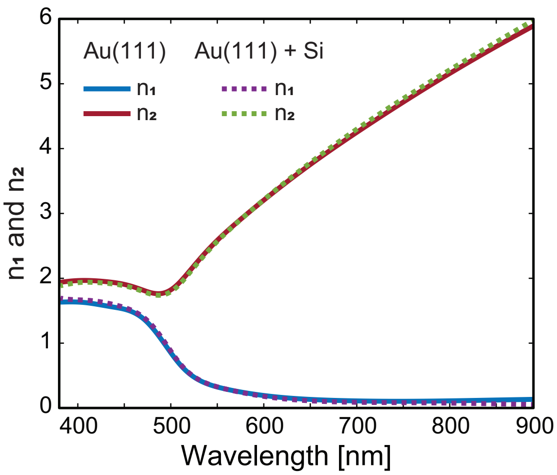

Figure S3: Refractive index (n_1) and extinction coefficient (n_2) of the bare Au(111) surface (blue and red solid line) and the same Au(111) substrate covered by 1 ML of degraded silicene (purple and green dashed line).

The optical properties of the Au(111) substrate, with and without a degraded silicene layer, were investigated in regards to its reflectivity and optical constants. The spectral reflectance curves (figure S2) were measured with a Filmetrics F20 thin-film measurement system and the refractive index and extinction coefficient (figure S3) were determined using a J. A. Woollam $\alpha - SE$ ellipsometer.

3. In-situ passivation

The hBN crystals are first mechanically exfoliated via a polyimide tape with a silicon adhesive suitable for UHV applications (figure S4 (a)). The tape is then mounted onto a spring-loaded cylinder on a custom designed UHV-compatible stage (figure S4 (b)). The stage is then placed inside a separate UHV chamber adjacent to the growth chamber. A bake-out step is performed after the insertion of the tape, to remove adsorbed species from the surface of the exfoliated flakes. After the silicene growth, the Au(111) sample is transferred into the stamping chamber onto the sample holder facing towards the exfoliated flakes (figure S4 (c)), whereby the position of the sample holder can be precisely controlled in x, y and z directions. The encapsulation is performed after cooling the sample down to a temperature

of approximately 80−100 °C. The spring-loaded cylinder promotes uniform contact of the tape to the sample and softens the stamping procedure to prevent damages to both the silicene layer and the substrate.

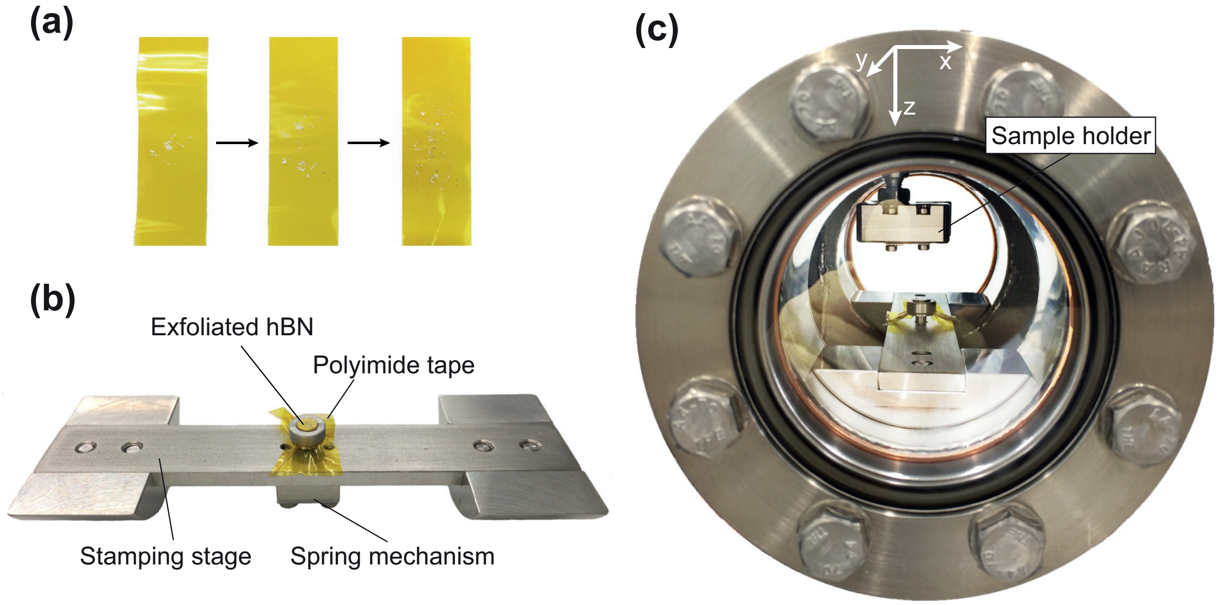

Figure S4: In-situ passivation methodology. (a) Mechanical exfoliation of hBN using polyimide tape. (b) Stamping stage loaded with the exfoliated hBN flakes. (c) Stamping apparatus inside the UHV system with the mounted stamping stage and the growth substrate above.
